# Immediate Space Closure and Alveolar Bone Regeneration Through Autotransplantation: A Case Report

**DOI:** 10.7759/cureus.97310

**Published:** 2025-11-20

**Authors:** Stratos Vassis, Jason Liu, Oskar Bauss

**Affiliations:** 1 Section of Orthodontics, Department of Dentistry and Oral Health, Aarhus University, Aarhus, DNK; 2 Orthodontics and Dentofacial Orthopedics, Orthodontic Private Clinic, Hannover, DEU

**Keywords:** bone defect regeneration, root development, root formation, space closure, tooth autotransplantation

## Abstract

This case report presents the clinical and radiographic outcome of immediate autotransplantation of a mandibular third molar into an infected first molar socket, with simultaneous space closure and alveolar bone regeneration. An 18-year-old female patient presented with a severely compromised right mandibular first molar due to a periapical lesion. The tooth was extracted, and the mandibular left third molar, selected for its favorable root development stage, was transplanted into the infected site during the same surgical session. Post-operative derotation was initiated with aligners and completed with segmental fixed appliances. One year after transplantation, cone-beam computed tomography showed complete bone healing and root maturation of the donor tooth. Clinically, the tooth was functional, sensibility returned during orthodontic treatment, and no ankylosis or root resorption was observed. Autogenous tooth autotransplantation in infected sites can preserve alveolar bone and support root development. However, it may cause intraoperative pain and require subsequent orthodontic treatment, which should be discussed preoperatively.

## Introduction

Autogenous tooth autotransplantation (ATT) is a surgical procedure involving relocation of a healthy tooth from one site to another within the same individual, most commonly using third molars or premolars as donor teeth ​​[1]. ATT offers an alternative to dental implants or fixed prostheses [2]. In adolescents, it offers cost-effectiveness and biological compatibility for tooth replacement, the potential for pulp regeneration in immature roots, and preservation of the alveolar bone [3]. ATT is especially indicated in cases requiring replacement of first or second molars due to factors such as non-restorable caries, trauma, periodontal disease, endodontic failure, or congenital absence [4]. It enhances masticatory function and maintains occlusal stability and dental arch integrity [5]. Long-term studies have demonstrated high survival rates, reaching up to 97.9%, with 1-, 5-, and 10-year survival rates reported at 98%, 95.9%, and 96.9%, respectively [6]. Notably, survival outcomes tend to be higher in children and adolescents (95.6%) compared to adults (80.3%), and single-rooted teeth generally show better outcomes than molars [6].

The determinant of ATT success is the donor’s root development stage. Optimal timing for transplantation is when root formation is approximately 75% complete and the apex remains open, facilitating revascularization and apexification [7]. Revascularization potential is also influenced by pulpal canal dimensions, which affect the likelihood of pulp survival and continued root development. A healthy periodontal ligament (PDL) on the donor tooth further enhances outcomes, as PDL stem cells have osteogenic potential, supporting bone regeneration and maintaining alveolar integrity [2,8]. However, ATT is not without complications. Post-operative sequelae may include inflammatory responses, pulp necrosis, root resorption, ankylosis, and incomplete root development [7]. The condition of the recipient site, particularly when inflamed or infected, adds further complexity to treatment planning. Whereas some authors prefer a delayed, two-stage approach to minimize the impact of local inflammation [2], others have reported favorable outcomes with immediate transplantation, even into previously infected sites, suggesting that ATT may also facilitate the regeneration of bone lost due to periapical pathology [4]. Other studies have examined antibiotic prophylaxis in ATT. Barendregt and colleagues found higher survival rates when prophylactic antibiotics were included in the protocol [9].

This case report presents the immediate autotransplantation of a mandibular left third molar into an infected extraction socket of the mandibular right first molar. The objective of this case report is to assess clinical and radiographic outcomes of ATT in an inflamed recipient site.

This case report was previously presented at the 97th German Orthodontic Society Congress 2025 on September 18, 2025. 

## Case presentation

Diagnosis and etiology

**Figure 1:**
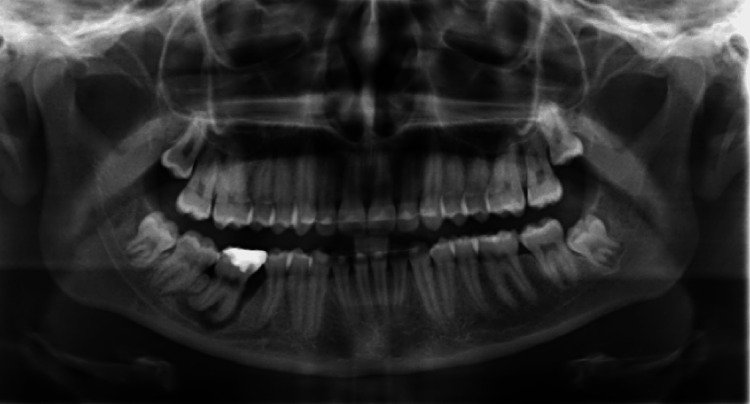
Panoramic radiograph The figure presents the panoramic radiograph. A clearly marked periodontal lesion is visible at the mandibular right first molar that also shows a large composite buildup. Fixed retainers are present from canine to canine in both arches, and mild crowding is observed in the mandibular posterior region.

An 18-year-old female patient presented herself to the orthodontic clinic in August 2024 following a one-year stay abroad. She had previously completed orthodontic treatment and was in retention with an upper Essix retainer and fixed retainers from canine to canine in both arches. A panoramic radiograph was obtained to assess the prognosis of the mandibular right first molar and revealed the presence of the lower wisdom teeth (Figure 1). The lower right molar showed a severe periapical lesion, necessitating its extraction.

**Figure 2:**
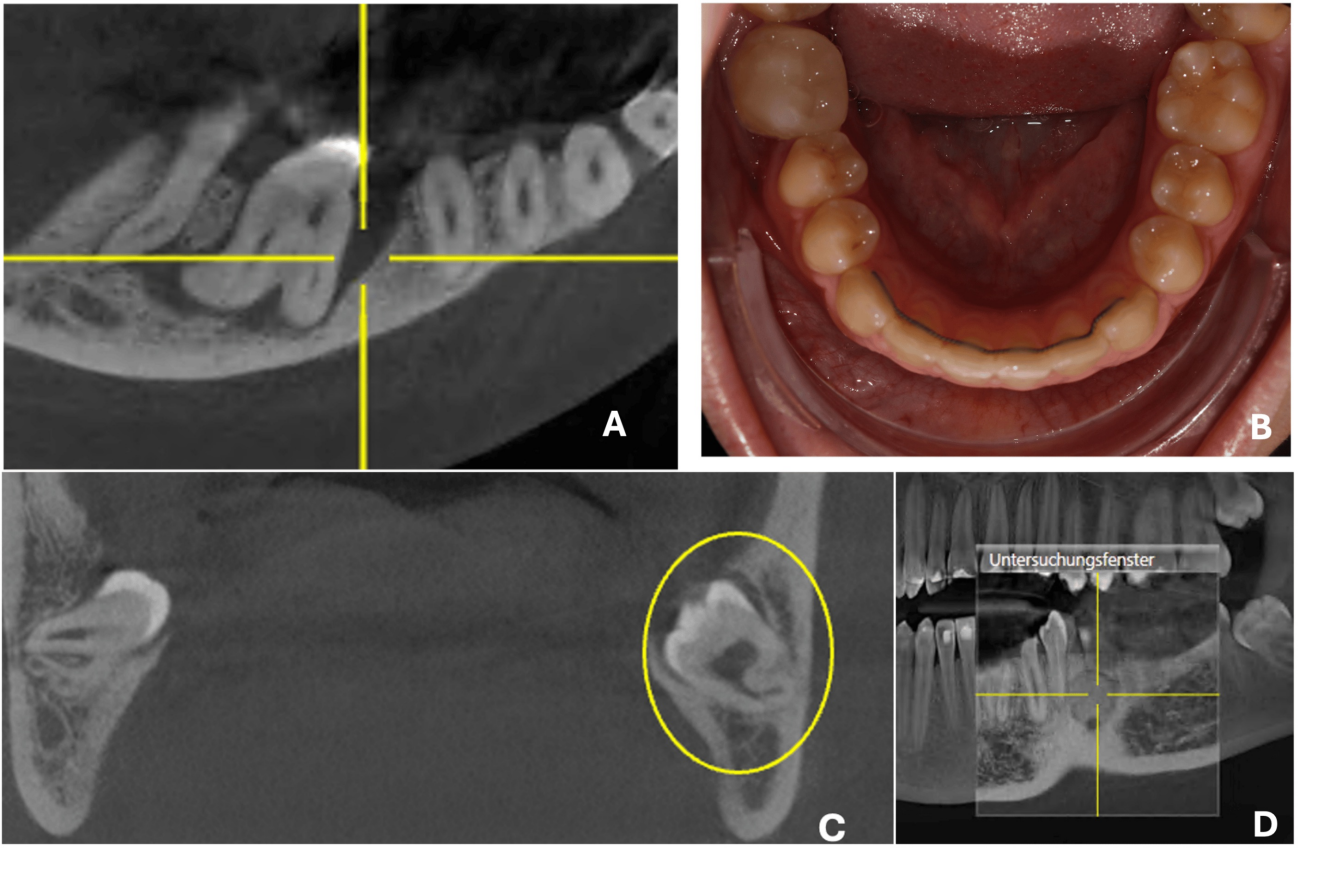
CBCT view of infectious site and donor tooth options Figure 2 shows (A) the extent of the infection of the lower right molar on the CBCT scan, (B) the intraoral photograph of the lower arch, (C) both wisdom teeth, from which the lower left was selected as the donor tooth due to its less advanced root formation, and (D) an incidental finding of a spherical radiopaque mass in the region of the lower left premolars. CBCT, cone-beam computed tomography.

A sectional cone-beam computed tomography (CBCT) scan of the mandible, excluding the condylar region, was obtained to further assess the site of infection (Figure 2A) and to identify the most suitable third molar donor based on root development stage (Figure 2C). The lower left third molar was selected as the donor tooth due to its open apical foramen and a slightly less advanced root formation stage compared to the lower right third molar. Additionally, selecting the lower left third molar provided a secondary treatment plan option: if the autotransplantation were to fail, space closure using temporary anchorage devices could be performed, allowing the second molar to be moved into the first molar position and the third molar into the second molar position. The CBCT report also revealed an incidental finding characterized by a well-defined, spherical radiopaque mass in the region of the lower left premolars (Figure 2D). 

Treatment objectives

The treatment objectives were to extract the lower right first molar and autotransplant the lower left third molar as the donor tooth, aiming for immediate space closure. Root development as well as periodontal and osseous healing were monitored, with radiographic follow-up. 

Treatment progress

**Figure 3:**
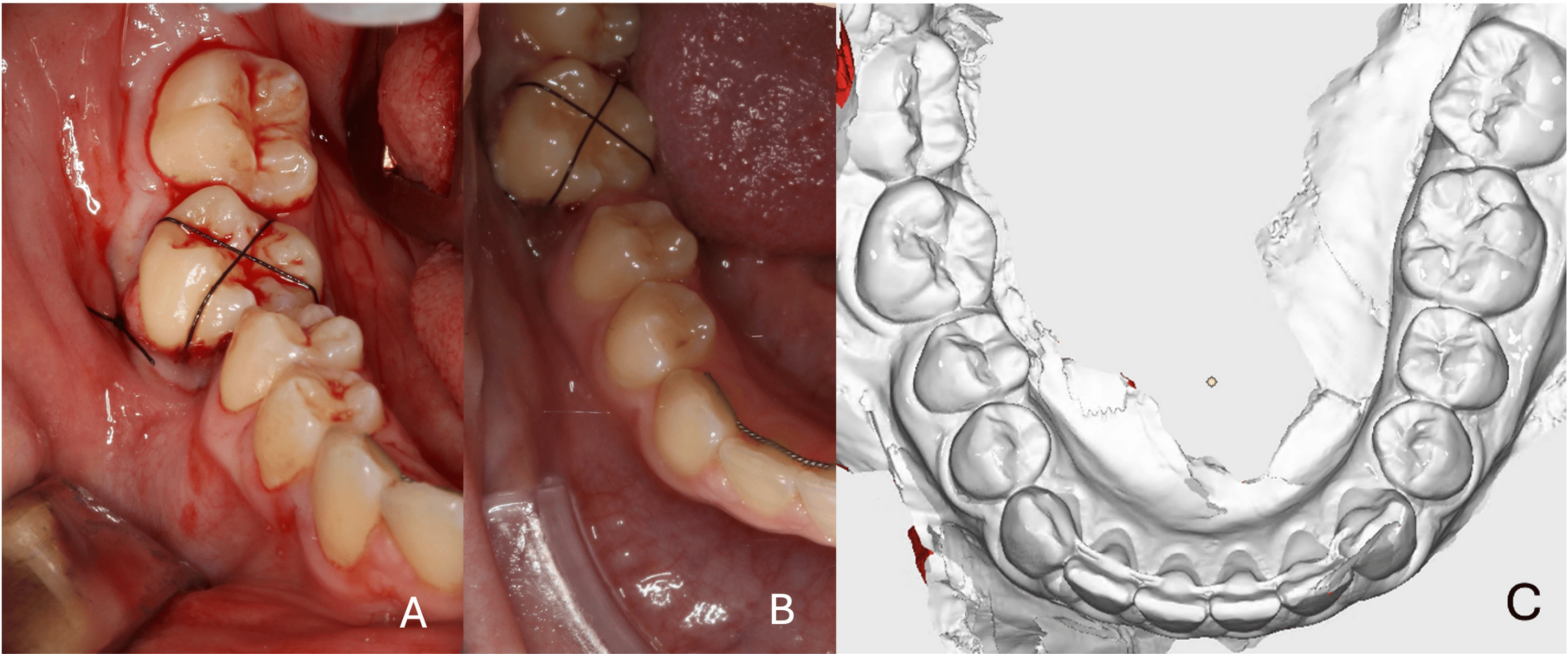
Post-surgical situation Figure 3 shows (A) the intraoral view immediately after autotransplantation, (B) the post-operative intraoral situation two days later, and (C) the initial intraoral scan for the planning and manufacturing of direct-to-print aligners.

In a single surgical session, the mandibular right first molar was extracted, the inflammatory cystic cavity was prepared, and the mandibular left third molar was autotransplanted and stabilized using a cross-stitch suture. Due to the severity of the infection and insufficient anesthetic efficacy in the affected region, both the extraction and the preparation of the cystic cavity were challenging. Patient reported pain and discomfort during surgery, and the donor tooth was placed rotated into the cystic cavity (Figure 3). Postoperatively, the patient was prescribed amoxicillin to be taken three times daily for seven days.

Due to the rotated positioning of the donor tooth, a follow-up consultation with the patient was scheduled two days after autotransplantation. It was recommended to allow a healing period of six months before initiating derotation using direct-to-print aligners. The lower fixed retainer was maintained, and the treatment goal was limited to align the molar without the use of attachments. During the course of aligner therapy, the patient reported wearing the appliance for less than 10 hours per day, resulting in suboptimal tooth movement. Consequently, treatment was converted to a segmental full fixed appliance approach to complete the derotation. A radiographic image was taken after surgery, and a one-year follow-up has been planned.

Results

**Figure 4:**
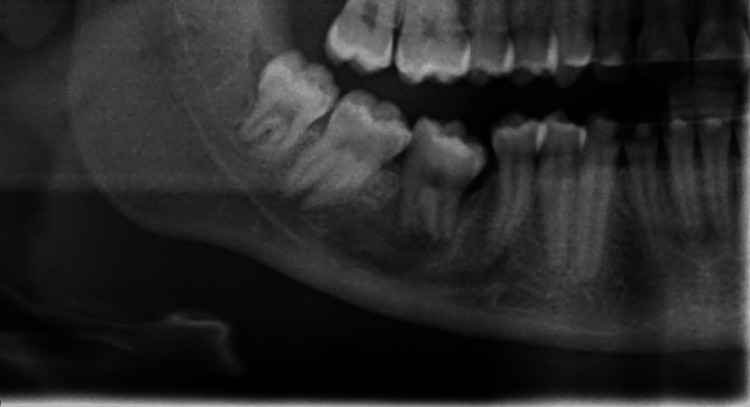
Post-operative sectional panoramic radiograph The figure illustrates the ATT in its final position, with both root apices remaining open. The open apex of the distal root was in close mesial contact with bone, and a mesial space was noticeable. The tooth was positioned directly into the extraction alveolus. ATT, autogenous tooth autotransplantation.

Following surgery, a sectional panoramic radiograph was obtained to evaluate the final positioning of the autotransplanted tooth (Figure 4).

**Figure 5:**
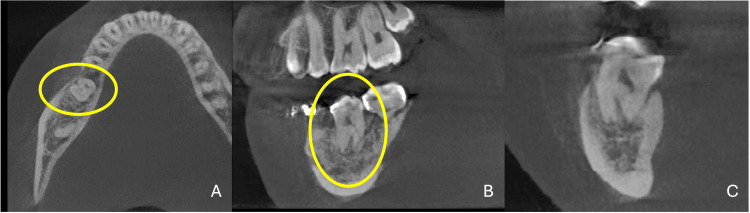
Sectional CBCT of the donor tooth at one year follow-up Figure 5 (A) presents the donor tooth from the coronal view and (B and C) illustrate the root development from the sagittal and coronal views. CBCT, cone-beam computed tomography.

**Figure 6:**
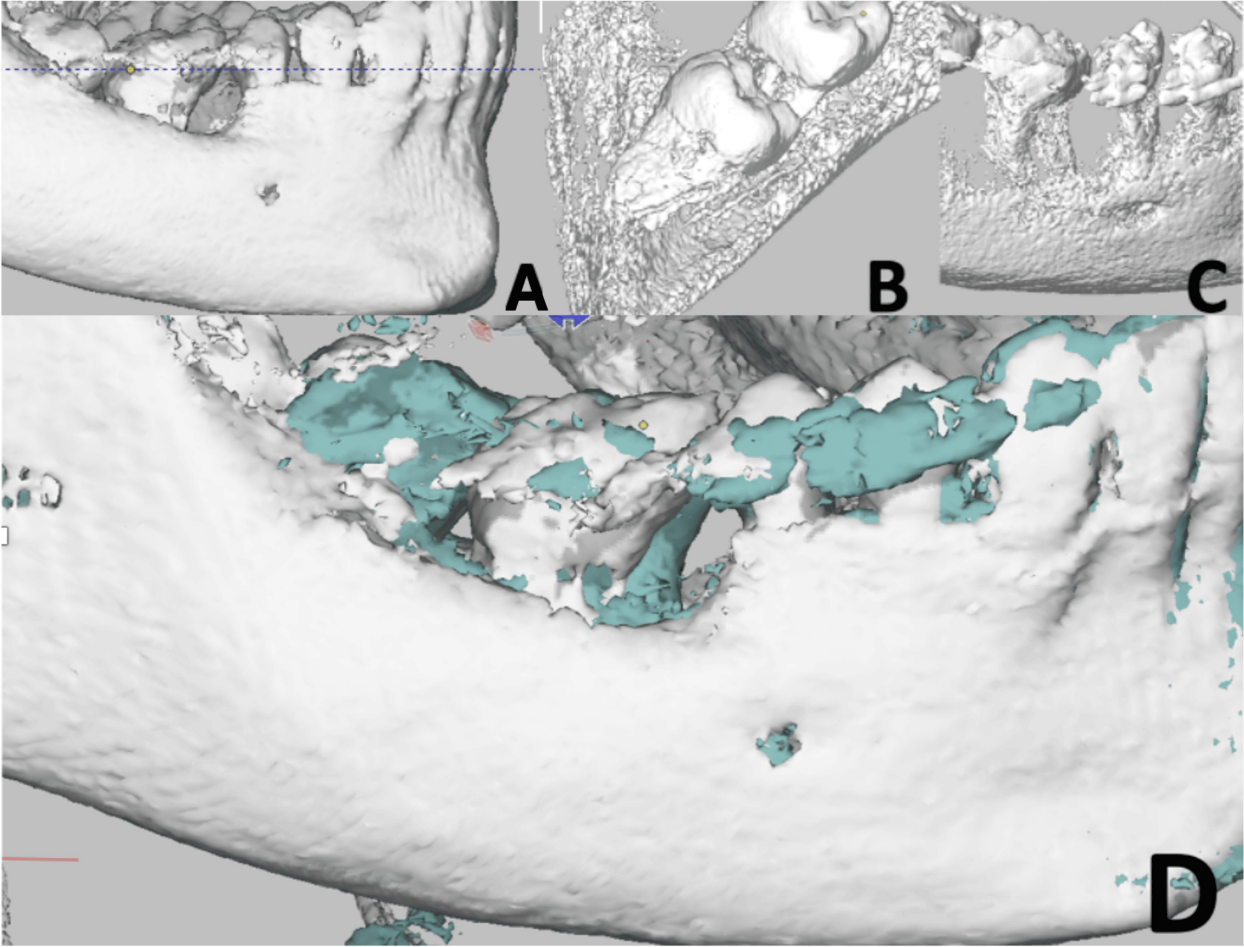
CBCT superimposition Figure 6 shows (A) the mandibular first molar presenting with a bony lesion, (B) the donor tooth in its original position, (C) the donor tooth in its final transplanted position, and (D) the superimposition of the initial CBCT scan with the one-year follow-up scan, aligned on the mandibular incisors, where white indicates the pre-operative image and green represents the post-ATT image. ATT, autogenous tooth autotransplantation; CBCT, cone-beam computed tomography.

The donor tooth was not ankylosed on examination, and the patient was able to function well. Sensibility testing was negative during the observation period but had shown a positive response during the derotation phase with full fixed appliances. A sectional CBCT scan of the mandible, excluding the condylar region, was performed one year after ATT. Sectional CBCT examination was performed not only to monitor root maturation but also to assess the previously identified spherical hard-tissue mass located interproximally between the lower left premolars (Figure 5). Superimposition of the initial scan with the scan obtained one year after autotransplantation demonstrated positional changes between the transplanted donor tooth and the original first molar at that site, in addition to evidence of bone remodeling (Figure 6).

## Discussion

This case report describes the successful immediate autotransplantation of an unerupted and immature mandibular third molar to replace a severely compromised first molar, extracted due to extensive pathology. Immediate ATT offers the significant advantage of space closure in a single surgical event; however, clinical challenges remain, especially in achieving adequate anesthesia at infected sites. This difficulty highlights the importance of pre-operative counseling to ensure patient understanding and cooperation [2]. In cases with extreme cystic lesions, patients should also be informed regarding the potential need for subsequent orthodontic treatment. It is important to consider that individuals who have previously undergone orthodontic therapy and received an ATT may exhibit reduced compliance with retreatment, which should be included during treatment planning.

Successful ATT is typically defined both radiographically by the presence of a continuous lamina dura and absence of root resorption or ankylosis and clinically by the lack of infection, discomfort, or pathological mobility [10]. In general, the success rate of autotransplanted immature third molars is lower than that of premolars at an equivalent stage of root development [11]. Several studies have identified optimal timing for autotransplantation to be when root formation has reached approximately one-half to three-quarters of its final length. This evidence served as the primary rationale for selecting the mandibular left third molar as the donor tooth, as it exhibited a more favorable root development stage than its contralateral [12,13]. Radiographic indicators of pulp vitality following autotransplantation include pulp canal narrowing or obliteration and/or continued root development. In the present case, progressive root development was observed, and sensibility testing during active treatment yielded a positive response, confirming successful revascularization and maintenance of pulp vitality [14].

Beyond preserving the vitality of the donor tooth, successful ATT in immature teeth contributes to alveolar bone preservation and growth, which is particularly beneficial in adolescent patients [11]. Consistent with this, the one-year follow-up CBCT scan demonstrated both continued root development and maintenance of alveolar bone structure (Figure 5). Overall, the follow-up scan revealed progressive root development and regression of the hard tissue mass.

A limitation of this case is the inadequate space available for the donor tooth, highlighting the need for pre-orthodontic treatment. Furthermore, additional cavity preparation would have been required, as the distal root of the donor molar was positioned in close contact with the mesial bony surface, thereby constraining the possibility of achieving an ideal three-dimensional placement. Post-orthodontic treatment might have been initiated earlier, as evidence supports starting tooth movement about two weeks after surgery [15]. However, the extensive bony defect resulted in limited initial stability, so we opted for a six-month period of masticatory stimulation to promote PDL healing.

The relationship between pre-existing periapical pathology and ATT outcomes remains complex. Although acute infection can compromise healing, the presence of a chronic periapical lesion may not always predict failure. In fact, an angiogenic environment during healing could enhance revascularization of both the pulp and PDL, as long as the site is adequately debrided and infection is controlled. This could explain the success reported in cases involving periapical lesions, including the present case [2,16]. However, due to variability in clinical presentations and the multifactorial nature of ATT success, further prospective studies with larger sample sizes are needed to better understand the impact of periapical pathology and to optimize patient selection and treatment protocols.

## Conclusions

ATT remains a biologically favorable treatment option in adolescent patients, especially when immature donor roots allow for revascularization and continued root development. In the present case, immediate ATT into an infected site resulted in continued pulp vitality and maintenance of alveolar bone, despite initial surgical complexity. This supports the concept that, under adequate debridement and infection control, periapical pathology alone is not a strict contraindication. However, infection at the transplantation site may cause intraoperative pain and discomfort and increase the likelihood of subsequent orthodontic treatment. These aspects must be clearly discussed with the patient before surgery.
